# Effects of Perch on Productivity, Welfare, and Physiological Indicators of Broiler Chickens Reared in Animal Welfare-Certificated Farms

**DOI:** 10.3390/vetsci11120614

**Published:** 2024-12-02

**Authors:** Byung-Yeon Kwon, Seong-Taek Kim, Da-Hye Kim, Jina Park, Hyun-Gwan Lee, Yong-Sung Jeon, Ju-Young Song, Sang-Ho Kim, Dong-Wook Kim, Chan-Ho Kim, Kyung-Woo Lee

**Affiliations:** 1 Department of Animal Science and Technology, Sanghuh College of Life Sciences, Konkuk University, Gwangjin-gu, Seoul 05029, Republic of Korea; byung64@konkuk.ac.kr (B.-Y.K.); seongtaek_0909@naver.com (S.-T.K.); kdh142536@naver.com (D.-H.K.); jinaa97@konkuk.ac.kr (J.P.); leehyun3177@naver.com (H.-G.L.); fmdyd94@kakao.com (Y.-S.J.); 0jysong970@gmail.com (J.-Y.S.); 2 K-AniWel, Gwonseon-gu, Suwon 16672, Republic of Korea; kims2051@gmail.com; 3 Department of Livestock, Korea National University of Agriculture and Fisheries, Deokjin-gu, Jeonju 54874, Republic of Korea; poultry98@korea.kr; 4 Animal Welfare Research Team, National Institute of Animal Science, Iseo-myeon, Wanju 55365, Republic of Korea; kch8059@korea.kr

**Keywords:** environmental enrichment, corticosterone, litter quality, welfare indicators

## Abstract

**Simple Summary:**

This study investigated the effects of perch provision on the productivity and welfare of broilers in two commercial farms. Broiler houses from each farm were provided with or without perches. The results showed that perches did not consistently affect productivity measures such as weight or uniformity. However, corticosterone levels, as the stress indicator, were lower in the group with perches. While there were no significant differences in some animal welfare indicators, gait scores were affected by perches in one of the farms employed. In conclusion, perches did not significantly improve broiler productivity or welfare, but they did have a positive effect on reducing stress. These findings provide important insights for improving broiler farming environments.

**Abstract:**

This study explored the impact of perches on the productivity and welfare of broilers raised on two animal welfare-certified farms (designated as Farm A and B) in South Korea. Broiler houses in each farm were provided with or without wooden square-shaped perches (2 × 2 cm) at a rate of 2 m per 1000 birds. The study aimed to assess whether perches could influence productivity measures, such as weight and uniformity, and animal welfare indicators, including corticosterone levels and physical health markers. The findings showed that the effects on productivity were inconsistent, varying by farm and period. Corticosterone levels, as an indicator of stress, were significantly lower in the perch group on farm B, but not on farm A. There were no significant differences in welfare indicators such as footpad dermatitis or feather cleanliness, although gait scores improved in farm B with perch provision. Litter moisture was higher in the perch group of farm A, but showed no difference in farm B. The study concluded that while perches did not consistently improve productivity or welfare, they did help reduce stress in broilers, as indicated by lower corticosterone levels.

## 1. Introduction

A perch is usually a tall structure that birds can grasp using their feet [1]. The behavior of chickens sitting on perches is a unique action that comes from their natural ancestors, and chickens prefer to perch on branches or perches at night [2]. This behavior is understood as an anti-predatory behavior in wild birds, as perching on high perches reduces the risk of capture by terrestrial predators [3,4]. This intrinsic behavior is still observed in chickens after domestication [5,6,7]. Animal welfare refers to providing animals with all required components to ensure physical and mental health [8]. Therefore, providing perches for broilers is important from an animal welfare perspective. In South Korea, animal welfare-certified farms for broilers must comply with the provision of perches within houses.

Broilers generally use fewer perches than laying hens, possibly because of their heavier body weight [9,10,11]. The frequency of perch use by broilers is related to the genotype associated with growth rate, with slow-growing breeds using perches more than fast-growing breeds [12,13]. The use of perches by broilers not only satisfies instinctive behavioral motivation and improves physical development but also provides an opportunity to regulate body temperature from heat stress away from the floor in a high-temperature environment [6,14]. Studies on the effect of stocking density, perch material, and height on perch use and broiler chickens have been conducted [10,15,16], but research on commercial broiler farms, which generally provide single perches of wooden square bar in South Korea, is lacking.

Thus, this study aimed to study the effect of perch provision on the productivity and indicators for animal welfare and physiology of broilers in animal welfare-certified broiler farms. Two animal welfare-certified farms at different locations were selected and several farm visits were made to evaluate the perch provision. A perch was provided to the houses, and the perch effect, if any, was analyzed based on welfare status indicators. The data obtained from this study can be used to improve our understanding of the basis of animal welfare in the commercial broiler farms. Indicators of broiler welfare analyzed in this study have been well acknowledged and highlighted elsewhere [17].

## 2. Materials and Methods

All experimental protocols and use of broilers in the trial were approved by the Institutional Animal Care and Use Committee of Konkuk University (KU21208-1).

### 2.1. Birds and Experimental Farms

The experiment was studied at two welfare-certified animal farms (Table 1) [18]. The broiler welfare-certified farms in Korea are officially required to maintain low stocking density (less than 19 broilers and less than 30 kg/m^2^) and to provide perches, minimum 6 h continuous darkness in lighting, fresh air, and plant-based nutrient adequate and balanced diets at all stages of production. The first farm (A), located in Boseong province, had five broiler houses and two out of the five houses in Farm A were selected. Each house was 120 m × 16 m (length × width; area, 1920 m^2^) and housed approximately 32,000 birds (stocking density was 16.7 birds/m^2^). Rice hulls are used as litter, and fresh litter is replaced each time broiler chickens are marketed. The second farm (B), located in Yeongam province, had five broiler houses and two out of the five houses in Farm B were selected. Each house was 100 m × 16 m (length × width; area, 1600 m^2^) and housed approximately 26,800 birds (the stocking density was 16.8 birds/m^2^). In addition, rice hulls were used as litter, but unlike in farm A, they were fermented and recycled for every six flocks.

Both farms raised the commercial broiler strain (unsexed Arbor Acres) supplied by an integrated company. Water and feed were provided ad libitum. As a feed, the early and late phase feeds mainly formulated with corn and soybean meal were supplied, and the formula ratio and ingredients were not presented for the security of the integrated company. The crude protein levels in starter and finisher diets were analyzed to contain 22.0% and 19.1% in Farm A and 22.2% and 19.0% in Farm B, respectively. The photoperiod was gradually changed from 23 L:1D upon chick placement to 18 L:6D on day 5 post-hatch and kept constant until day 28.

### 2.2. Perch

**Figure 1:**
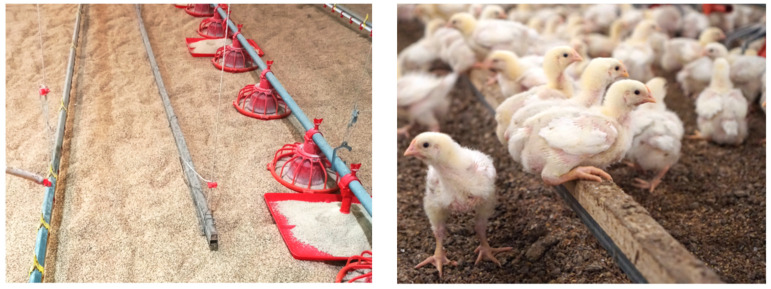
The figure of perch used in the experiment.

The perch used in this experiment was a wooden square bar with a thickness of 2 × 2 cm, which was supported by a metal bar underneath to support the weight of the birds (Figure 1) and was placed at the rate of 2 m per 1000 birds. The height was adjustable; therefore, it was adjusted to the height of the neck between the head and back. The perch was installed between the water nipple line and feeder line, evenly at regular intervals, with a total of two lines, one line on each side positioned at the center of the house.

### 2.3. Research Schedule and Measurements

#### 2.3.1. Research Schedule

**Figure 2:**
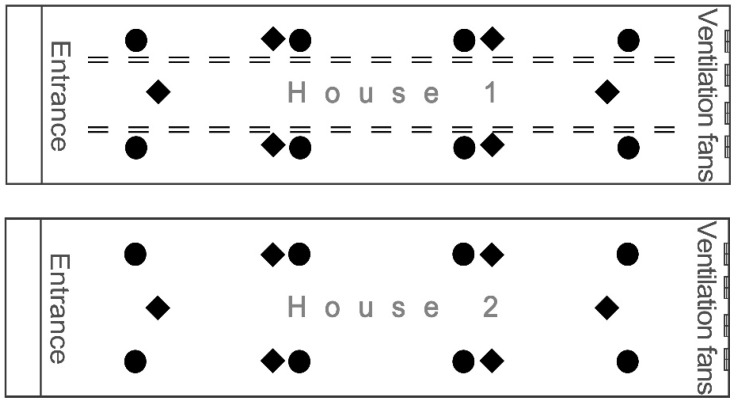
The sampling locations of parameters and the perch placement in houses. The symbol equal (=) represents the perch, while the black diamonds and the black circles denote sampling locations. The black diamond symbols indicate locations for body weight measurement, and the black circle symbols indicate locations for monitoring feathers and fecal corticosterone, footpad dermatitis, hock burn, feather cleanliness, litter, and body surface temperature.

Multiple farm visits on Farm A and a single farm visit on Farm B were made to collect data or samples for productivity, litter, and welfare indicators. The evaluation measurements during farm visits were performed at 26 days of age for each farm. Hatched chicks were introduced in the autumn season of animal welfare-certified farm A on 4 October 2021 and were investigated on October 29. The spring season was investigated twice; hatched chicks were introduced on 18 March 2022, and investigated on April 12, and then introduced again on 7 May 2022, and investigated on June 1st. Animal welfare-certified Farm B was investigated once in spring, and hatched chicks were introduced on 9 May 2022, and investigated on June 3. Broiler welfare evaluation parameters and sample numbers are presented in Table 2 and Figure 2.

#### 2.3.2. Productivity

The body weight and flock uniformity of 26-day-old birds were investigated. The body weights of 10 broilers were measured (SWII-W, CAS Co., Seoul, Repubic of Korea) at six locations for each treatment (10 birds/location × six locations × one house), and the coefficient of variation (CV) was calculated for each location.

#### 2.3.3. Corticosterone

Sampling freshly voided fecal droppings (five pieces/location × four locations × two houses) and feathers (0.05 g of feathers collected from the interscapular area per broiler) (five birds/location × eight locations × one house) at 26 days were described elsewhere and analyzed for corticosterone concentrations [18,19]. No attempts in samplings were made to distinguish male and female broilers, but those close to flock mean body weight were sampled to eliminate to some extent the misinterpretation resulting from sex differences between sampling locations. The feather and fecal samples were analyzed for corticosterone using a commercial corticosterone ELISA kit (Catalog No. ADI-901-097; Enzo Life Sciences, Farmingdale, NY, USA), according to the manufacturer’s specifications. The absorbance was measured at 405 nm using a spectrophotometer (Synergy 2; BioTek Instruments, Inc., Winooski, VT, USA).

#### 2.3.4. Determination of Animal Welfare Indicators

Upon farm visits (at 26 days), animal welfare indicators (that is, footpad dermatitis, hock burn, and feather cleanliness) were assessed as described earlier [18,19] according to the Welfare Quality^®^ Assessment protocol for poultry [20].

#### 2.3.5. Gait Score

The gait score as an additional animal welfare indicator was assessed on a three-point scale per the protocol [21] as described earlier [17,18].

#### 2.3.6. Litte Quality

Litter samples (100 g per location) were collected from eight locations in each treatment (one sample/location × eight locations × one house). The litter (approximately 50 g) was dried at 135 °C for 2 h and the nitrogen in the litter was analyzed using the Kjeldahl method. pH of the litter was analyzed using a pH meter (Lab 845, SI analytics, Mainz, Germany) by mixing 2 g of the litter sample with 40 mL of distilled water according to the method of Coufal et al. [22].

#### 2.3.7. Body Surface Temperature

Upon farm visit (at 26 days), body surface temperature was monitored as described elsewhere [18,19]. In brief, 80 birds at eight locations per treatment group were measured using a thermographic camera (E8-XT, FLIR System, Wilsonville, OR, USA).

### 2.4. Statistical Analysis

Replicates of this experiment were performed at the sampling locations for each treatment. The Student *t*-test was used to analyze the data collected from the survey at the significance level of 5% using SAS (v9.4; SAS Institute Inc., Cary, NC, USA). A chi-square test or Fisher’s Exact Test was performed for the animal welfare data (i.e., footpad dermatitis, hock burn, feather cleanliness, and gait score) expressed with frequency and ratio.

## 3. Results and Discussion

### 3.1. Body Weight and Uniformity

The effects of perches on body weight and flock uniformity in broilers were presented (Table 3). In Farm A, body weight in the perch treatment group was significantly higher during March to April (*p* < 0.001) but lower during May to June (*p* < 0.001) compared to that in the control group. In Farm B, body weight in the control vs. perch group was heavier during May to June (*p* = 0.043). Collectively, the effect of perch on body weight was inconsistent, and no significant difference was observed in the uniformity of body weight between the perch treatments during all experimental periods.

Earlier studies on the effect of perches on body weight have reported no effect on body weight [23,24,25,26,27,28]. The purpose of using a perch when rearing broilers is to satisfy behavioral needs and to relieve stress for farm animal welfare, rather than improving productivity. According to a review by Riber et al. [14], as a type of environmental enrichment, the perch was evaluated as a point-source enrichment suitable for satisfying the motivation of broilers to rest in a high structure without affecting productivity. Therefore, the inconsistent results on body weight shown in this study might be influenced by other uncontrolled fixed variables due to the characteristics of the experimental site where approximately 20,000 to 30,000 commercial broilers are raised per house. However, studies have shown that a cool perch is effective in increasing body weight [10,29,30]; therefore, additional research is needed to evaluate productivity and welfare improvement applicable to broiler farms.

### 3.2. Corticosterone Concentration

Table 4 shows the effects of perch on corticosterone levels in feces and feather samples. Perching did not affect fecal and feather corticosterone in farm A, but it lowered fecal corticosterone levels in Farm B compared to the no-perch control groups.

The low fecal corticosterone level in the perch treatment group of Farm B suggests that the perch may be effective in reducing broiler stress. It remains unclear why perch provision did not affect fecal corticosterone on Farm A, although both farms received equal management, including diet and stocking density. Owing to the lack of studies evaluating the effect of perch on corticosterone in broiler chickens, the perch-mediated decrease in corticosterone concentration is yet to be determined. However, Alm et al. [31] emphasized that caution should be exercised when interpreting the results because various factors, such as the fiber content in feed and excretion rate, may affect fecal corticosterone metabolites.

### 3.3. Animal Welfare Indicators

Table 5 shows the effect of perch on footpad dermatitis, hock burn, and feather cleanliness in broiler chickens. There were no significant differences in the observed parameters in broilers provided with or without a perch.

Footpad dermatitis and hock burns are closely related with wet litter status and heavier broiler weight [32,33,34,35]. In this experiment, it was expected that the perch would prevent contact dermatitis, such as footpads and hocks, and improve feather cleanliness, because the perch keeps the broiler body away from the floor litter. De Jong et al. [36] reported that providing an enrichment consisting of a perch and platform did not affect the prevalence of contact dermatitis or walking ability in broilers, and Vasdal et al. [7] also reported no significant perch effect on footpad dermatitis, breast blisters, and keel bone damage in broiler breeders. In contrast, Kiyma et al. [37] reported that perches had a positive effect on footpad dermatitis prevention in broilers. Ventura et al. [38] also reported that footpad dermatitis was improved when a simple barrier perch was used in broiler rearing. Oester and Widmer [39] reported fewer hock burns when using a ramped platform and an elevated perch. Other studies on perches have shown inconsistent effects on footpads and hock dermatitis. These differences may be related to the age and body weight of the birds, the frequency of perch use, and the degree to which the birds are encouraged. Furthermore, these differences may be related to the type of perch configuration and the difference in experimental design. In any event, the current study showed that the perch per se did not affect welfare indicators (that is, footpad dermatitis, hock burn, and feather cleanliness), indicating that broilers reared in the current environments, irrespective of perch, performed and managed well.

### 3.4. Gait Score

Table 6 shows the results of the assessment of the effect of the perch on the gait scores of broilers at 26 days of age. It was found that the perch did not affect the gait score in Farm A but increased it in Farm B.

The gait score of broilers is an indicator of leg health (e.g., leg weakness or lameness), along with footpad dermatitis and hock burns [40,41]. Factors affecting the gait score include growth rate by breed, age, body weight, and stocking density [42]. De Jong et al. [36] reported that enriching perches and platforms does not affect broiler walking ability. Bailie and O’Connell [5] showed that providing a perch and pecking objects improved behavior and welfare without a clear effect on leg health. In contrast, Kaukonen et al. [43] reported that the ratio assessed as a gait score of 3 was significantly lower in the platform perch treatment; however, there was no difference between scores of 4 and 5. Thus, the lack of an effect of the perch on gait scores observed in Farm A can be expected, based on other studies. However, in contrast to expectations, gait scores were higher in the perch group in Farm B, indicating that the perch negatively affected leg health. Paz et al. [44] showed that reused litter has a higher prevalence of footpad lesions than fresh litter, which could lead to lameness in broilers. In contrast to the fresh litter in Farm A, the reused litter utilized in Farm B might have influenced the gait score, regardless of whether perches were present. However, it should be noted that only seven birds had gait scores of 4 and 5 in the perch group compared with the control group, which had no gait score above 4, reaching statistical significance between the treatment groups. This result may not be related to perch use by the affected broilers, which needs to be addressed further.

### 3.5. Litter Quality

Table 7 shows the effects of the perch on the moisture, nitrogen, and pH of the litter within broiler houses. The litter moisture in Farm A was higher in the perch group in autumn (October) (*p* = 0.048) and spring (May to June) (*p* = 0.020), and nitrogen content was significantly higher in the no-perch group in spring (*p* = 0.032). It was found that litter quality was not altered between treatments at Farm B.

Broiler litter is a mixture of excreta, beddings, feeds, and feathers [45]. The moisture level in the litter is often used as an animal welfare indicator because it affects footpad dermatitis and hock burn [46,47,48,49]. This study found that perch provision increased the moisture content in litters sampled during October and May to June, particularly in Farm A. However, the perch-mediated increase in litter moisture content did not affect contact dermatitis in this study. At this stage, no clear explanation is readily available. Thus, whether the perch per se increases water intake must be addressed.

The nitrogen in the litter increases with the accumulation of broiler manure and is released as ammonia through a series of mechanisms [50]. In this study, litter nitrogen content was higher in the control group than in the perch group when the litter was sampled during spring. The perch per se did not affect litter nitrogen or pH because other studies have shown that there is no correlation between the use of perches and litter quality during broiler rearing [51,52,53]. Whether perch provision increases or decreases feed and water intake must be addressed. The latter statement is considered important for disclosing perch-associated behaviors related to feed and water intake.

### 3.6. Body Surface Temperature

Table 8 shows the effects of perch on the body surface temperature. In Farm A, perch-provided broilers had higher (*p* < 0.05) body surface temperatures of the head, chest, and legs compared with the no perch-provided control group in October. However, no effect of perch on body surface temperature was noted when analyzed from March to April and May to June in Farm A and from May to June in Farm B.

The body surface temperature of broilers can be measured in a non-invasive manner to evaluate the state of body temperature regulation [54,55,56]. Yildirim and Taskin [57] reported that there was no difference in the rectal temperature between the untreated control and enriched treatment using perches and balls on days 21 and 42 of the controlled experiments. Zhao et al. [10] studied the effect of a water-chilled cool perch as a solution to high-temperature stress in broilers and found that the cool perch was effective in lowering rectal temperature in hot environments, improving welfare. As the current study was conducted in a commercial farm setting, the effect, if any, of the perch on body surface temperature was considered minimal. Instead, the analyzed body surface temperatures observed in this study could be secondary to the temperature and temperature–humidity index (THI) in the house when taking measurements, as recommended by [58]. However, monitoring the body surface temperature of broilers could be a useful tool for evaluating the welfare status of broiler flocks when their welfare is compromised or they are under heat stress.

## 4. Conclusions

It is concluded that the welfare status of broiler chickens was not clearly improved in broiler chickens raised with or without a perch. However, perch provision lowered fecal corticosterone, indicating the role of perch in relieving the stress response. Future studies are warranted to evaluate the behavior patterns of broilers on perch use in the commercial welfare-certified broiler farm settings.

## Figures and Tables

**Table 1:** Primary characteristics of the experimental farms.

Items	Experimental Farms	
	Farm A	Farm B
Farm location	Boseong, Jeollanam-do	Yeongam, Jeollanam-do
Strain	Arbor Acres	
House type	Windowless	
Ventilation type	Forced exhaust	
Flock size, number of birds	32,000	26,800
House size, m, m^2^	120 × 16 = 1920	100 × 16 = 1600
Stocking density, birds/m^2^	16.67	16.75
Litter type and replacement cycle	Fresh rice hulls	Recycled rice hulls
Perches installed	Wood, 2 m per 1000 birds	

**Table 2:** Broiler welfare assessment parameters and number of samples.

Measurement/Observation	Number of Samples
Corticosterone	Feathers and droppings were sampled at 8 locations in a treatment from 40 birds
Footpad dermatitis	Footpad pictures of 10 birds were taken at 8 locations in a treatment at 26 days
Hock burn	Hock pictures of 10 birds were taken at 8 locations in a treatment at 26 days
Feather cleanliness	Feather pictures of 10 birds were taken at 8 locations in a treatment at 26 days
Gait score	Broilers were made to slowly walk through 20% of the house area and the moving flock was recorded at 26 days
Litter	One sample of about 100 g was collected from 8 locations in a treatment at 26 days
Body surface temperature	At 8 locations in the house, 10 broilers on the 26th day, a total of 40 broilers, were photographed with a thermal imaging camera

**Table 3:** Body weight and uniformity of 26-day-old broilers ^1^.

Items	Treatment				*p*-Value
	Control		Perch		
	Mean	SD ^1^	Mean	SD ^1^	
Farm A					
Autumn (October)					
Body weight, g/bird	1212	15.4	1182	36.8	0.098
CV ^2^, %	3.26	0.63	3.77	1.10	0.345
					
Spring (March–April)					
Body weight, g/bird	1258 ^b^	13.2	1333 ^a^	27.7	<0.001
CV ^2^, %	2.999	0.69	3.916	0.89	0.073
					
Spring (May–June)					
Body weight, g/bird	1257 ^a^	19.4	1185 ^b^	26.9	<0.001
CV ^2^, %	4.08	0.66	3.66	0.97	0.394
					
Farm B					
Spring (May–June)					
Body weight, g/bird	1313 ^a^	26.9	1275 ^b^	19.6	0.043
CV ^2^, %	4.52	1.42	4.16	0.85	0.661

^a,b^ Means (6 replicates per treatment) with a different superscript differ (*p* < 0.05). ^1^ SD, standard deviation. ^2^ CV, coefficient of variation.

**Table 4:** Corticosterone concentrations in feces and feathers of broilers provided with or without perch ^1^.

Items	Treatment				*p*-Value
	Control		Perch		
	Mean	SD ^2^	Mean	SD ^2^	
Farm A					
Autumn (October)					
Feces, pg/mg	7.84	1.91	6.57	2.26	0.399
Feather, pg/mg	3.58	2.19	4.02	0.59	0.653
Spring (March–April)					
Feces, pg/mg	25.80	9.72	32.31	3.50	0.171
Feather, pg/mg	16.60	1.38	18.51	1.83	0.068
Spring (May–June)					
Feces, pg/mg	18.11	8.43	25.14	5.17	0.064
Feather, pg/mg	6.39	0.13	6.12	0.59	0.276
Farm B					
Spring (May–June)					
Feces, pg/mg	27.55 ^a^	2.50	20.90 ^b^	3.44	0.001
Feather, pg/mg	7.21	0.80	7.75	0.35	0.160

^a,b^ Means with a different superscript differ (*p* < 0.05). ^1^ Values are means of eight replicates per treatment. ^2^ SD, standard deviation.

**Table 5:** Percentage of footpad dermatitis, hock burn, and feather cleanliness score assessment results in broiler chickens provided with or without perch ^1^.

Items	Treatment						χ^2^ Test (*p*-Value)	Fisher’s Exact Test ^2^ (*p*-Value)
	Control			Perch				
	Score 0	Score 1	Score 2	Score 0	Score 1	Score 2		
Farm A								
Autumn (October) ^3^								
Footpad dermatitis	100	0	0	97.1	2.9	0	3.187 (0.074)	0.115
Hock burn	98.2	1.8	0	98.1	1.9	0	0.002 (0.963)	1.000
Feather cleanliness	87.3	12.7	0	86.7	13.3	0	0.017 (0.895)	-
Spring (March–April) ^4^								
Footpad dermatitis	99.0	1.0	0	98.1	1.9	0	0.358 (0.550)	0.620
Hock burn	94.3	5.7	0	97.1	2.9	0	0.986 (0.321)	0.498
Feather cleanliness	86.7	12.4	1.0	91.3	8.7	0	1.757 (0.415)	0.437
Spring (May–June) ^5^								
Footpad dermatitis	97.6	2.4	0	100	0	0	2.123 (0.145)	0.237
Hock burn	98.8	1.2	0	94.2	5.8	0	2.573 (0.109)	0.211
Feather cleanliness	76.8	19.5	3.7	84.9	15.1	0	3.953 (0.139)	0.138
Farm B								
Spring (May–June) ^6^								
Footpad dermatitis	97.5	1.3	1.3	94.2	4.7	1.2	1.642 (0.440)	0.683
Hock burn	93.8	6.3	0	95.3	4.7	0	0.207 (0.649)	0.740
Feather cleanliness	81.3	18.8	0	84.9	14.0	1.2	1.582 (0.453)	0.528

^1^ Values are percentage of assessed birds. ^2^ When at least one expected frequency in a table is less than 5, the Fisher’s Exact Test is more appropriate than the χ^2^-test. ^3^ n = 110 for control; n = 105 for perch treatment. ^4^ n = 105 for control; n = 103 for perch treatment. ^5^ n = 82 for control; n = 86 for perch treatment. ^6^ n = 80 for control; n = 86 for perch treatment.

**Table 6:** Percentage of gait score in broiler flocks provided with or without the perch ^1^.

Items	Treatment ^2^						χ^2^ Test (*p*-Value)	Fisher’s Exact Test ^3^ (*p*-Value)
	Control			Perch				
	Score 3	Score 4	Score 5	Score 3	Score 4	Score 5		
Farm A ^4^								
Autumn (October)	0.063	0	0	0.063	0.031	0.031	4.001 (0.261)	0.378
Spring (March–April)	0.063	0.047	0	0.078	0	0	3.111 (0.211)	0.385
Spring (May–June)	0.109	0.094	0.031	0.141	0.031	0.047	2.450 (0.484)	0.548
Farm B ^5^								
Spring (May–June)	0.075	0	0	0.131	0.075	0.056	7.828 (0.050)	0.035

^1^ Values are percentage of assessed 20% of the total number of birds. ^2^ Score 3, obvious abnormality, affects ability to move; Score 4, severe abnormality, only takes a few steps; Score 5, incapable of walking [20]. ^3^ When at least one expected frequency in a table is <5, the Fisher’s Exact Test is more appropriate than the χ^2^-test. ^4^ n = 6400 for control; n = 6400 for perch treatment. ^5^ n = 5360 for control; n = 5360 for perch treatment.

**Table 7:** Litter qualities in broiler chicken farms provided with or without perch.

Items	Treatment				*p*-Value
	Control		Perch		
	Mean	SD ^1^	Mean	SD ^1^	
Farm A					
Autumn (October)					
Moisture, %	27.9 ^b^	1.28	34.0 ^a^	3.56	0.048
Nitrogen, %	2.33	0.44	2.09	0.03	0.437
pH	7.26	0.06	7.26	0.06	0.939
Spring (March–April)					
Moisture, %	32.3	1.50	31.8	4.50	0.861
Nitrogen, %	2.24	0.08	2.30	0.12	0.527
pH	6.90	0.24	7.16	0.10	0.149
Spring (May–June)					
Moisture, %	23.1 ^b^	1.61	25.6 ^a^	2.04	0.020
Nitrogen, %	2.19 ^a^	0.14	2.03 ^b^	0.11	0.032
pH	6.79	0.20	6.88	0.14	0.326
Farm B					
Spring (May–June)					
Moisture, %	26.4	1.07	25.7	1.62	0.283
Nitrogen, %	2.73	0.07	2.68	0.08	0.221
pH	6.20	0.18	6.12	0.37	0.597

^a,b^ Means (eight replicates per treatment) with a different superscript differ (*p* < 0.05). ^1^ SD, standard deviation.

**Table 8:** Body surface temperature in broiler chickens provided with or without perch ^1^.

Items	Treatment ^2^				*p*-Value
	Control		Perch		
	Mean	SD ^3^	Mean	SD ^3^	
Farm A					
Autumn (October)					
Head, °C	37.4 ^b^	0.31	38.4 ^a^	0.93	0.048
Chest, °C	30.3 ^b^	0.75	32.8 ^a^	1.31	0.002
Legs, °C	35.8 ^b^	0.61	38.0 ^a^	0.58	<0.001
Temperature–humidity index	75.3		76.3		
Spring (March–April)					
Head, °C	38.7	0.81	38.1	0.74	0.250
Chest, °C	32.1	0.60	31.6	1.16	0.354
Legs, °C	38.6	1.48	37.7	0.97	0.269
Temperature–humidity index	77.6		77.9		
Spring (May–June)					
Head, °C	38.9	0.22	38.6	0.51	0.281
Chest, °C	32.1	0.35	31.7	0.61	0.114
Legs, °C	38.9	0.87	38.2	0.87	0.126
Temperature–humidity index	80.6		80.6		
Farm B					
Spring (May–June)					
Head, °C	39.0	0.22	38.9	0.56	0.557
Chest, °C	32.6	0.58	33.2	0.67	0.090
Legs, °C	39.6	0.30	39.7	0.62	0.638
Temperature–humidity index	89.8		88.6		

^a,b^ Means with a different superscript differ (*p* < 0.05). ^1^ Values are means of eight replicates. ^2^ Farm A, Autumn (October) − perch = temperature 25.3 °C, humidity 65.7%, THI 76.3; Autumn (October) − control = temperature 24.7 °C, humidity 68.3%, THI 75.3; Farm A, Spring (March–April) − perch = temperature 26.4 °C, humidity 57.3%, THI 77.9; Spring (March–April) − control = temperature 26.2 °C, humidity 59.4%, THI 77.6; Farm A, Spring (May–June) − perch = temperature 28.7 °C, humidity 29.6%, THI 80.6; Spring (May–June) − control = temperature 28.7 °C, humidity 29.4%, THI 80.6; Farm B, Spring (May–June) − perch = temperature 33.0 °C, humidity 40.6%, THI 88.6; Spring (May–June) − control = temperature 33.6 °C, humidity 40.9%, THI 89.8. ^3^ SD, standard deviation.

## Data Availability

Upon reasonable request, the datasets of this study can be available from the corresponding author.
